# The emergence of human influence on the ozone layer by the 1960s

**DOI:** 10.1073/pnas.2608286123

**Published:** 2026-06-29

**Authors:** Jian Guan, Benjamin D. Santer, Peidong Wang, Qiang Fu, Rolando R. Garcia, Yaowei Li, Kane Stone, Douglas E. Kinnison, Jun Zhang, Gabriel Chiodo, Jean-Francois Lamarque, Susan Solomon

**Affiliations:** ^a^ Department of Earth, Atmospheric, and Planetary Sciences, Massachusetts Institute of Technology, Cambridge, MA 02139; ^b^ School of Environmental Sciences, University of East Anglia, Norwich NR4 7TJ, United Kingdom; ^c^ Department of Atmospheric and Climate Science, University of Washington, Seattle, WA 98195; ^d^ National Center for Atmospheric Research, Boulder, CO 80305; ^e^ Instituto de Geociencias, Consejo Superior de Investigaciones Cientificas, Madrid 28040, Spain

**Keywords:** ozone depletion, early detection, CCl_4_

## Abstract

The Antarctic ozone hole was first reported in 1985, and small ozone losses at the global scale were also observed in the late 1980s. The combination of field and laboratory measurements, together with modeling, quickly established anthropogenic chlorofluorocarbons (CFCs) as the cause of both the Antarctic and global ozone depletion. However, when, where, and why the earliest ozone depletion could have been detected has not been determined. Here, we conduct a thought experiment to investigate when human-induced ozone depletion could have first been detectable, assuming the availability of accurate stratospheric ozone observations from 1950 onward. We find that human-caused ozone depletion was likely identifiable as early as 1957 in the tropical upper stratosphere. This region’s low internal variability enables the earliest detection of the anthropogenic signal, even though tropical ozone losses in the upper stratosphere were smaller than those in higher-latitude regions. Our results highlight the key role of considering both internal variability (“noise”) and the forced response (“signal”) in detection studies. Further, while CFCs are widely recognized as the primary drivers of current ozone depletion, we find that early ozone loss was primarily caused by human-made carbon tetrachloride (CCl_4_), used mainly as a solvent. These findings suggest that a clear human influence on the stratospheric ozone layer began nearly 70 y ago, even before substantial emissions of CFCs from spray cans or air conditioning.

## Significance

This paper examines the earliest emergence of human-caused ozone depletion: the when, the where, and the why. We employ a thought-experiment framework aimed at the physical emergence of the signal in a hypothetical world in which instruments had the same capabilities as today’s satellites. The “when” is as early as the late 1950s—about 30 y before the Antarctic ozone hole was discovered and 20 y before the Molina–Rowland theory. The “where” is the tropical upper stratosphere, where a relatively small signal stands out against even smaller noise, allowing the earliest emergence. The “why” is the use of carbon tetrachloride as a solvent decades before chlorofluorocarbons became common in refrigeration and spray cans.

The year 2025 marked the 40th anniversary of the discovery of the Antarctic ozone hole by Farman et al. in 1985 (1). This discovery was a shock to the scientific community and prompted research that elucidated its origins in heterogeneous chlorine chemistry (2). Ozone depletion by gas-phase chlorine chemistry in the upper stratosphere had been predicted about 10 y earlier (3), prompting many people and some governments to phase out the use of CFCs in spray cans by 1980. Upper stratospheric ozone depletion was subsequently reported in observations in the late 1980s (4, 5). The Montreal Protocol on ozone-depleting substances (ODS), adopted in 1987, is hailed as the world’s most successful environmental treaty to date (6). “World avoided” thought experiments estimate ozone depletion that would have happened in a world without the Montreal Protocol and have been useful for both science and science communication (7, 8). The Protocol will soon reach its own four decade mark amid signs of ozone recovery due to reduced emissions of ODS following its implementation (9–13). As this suite of landmark environmental events all reach 40th and 50th anniversaries in the 2024–2027 timeframe, it is timely to conduct a thought experiment (14) to probe when, where, and why the very first indicators of anthropogenic ozone depletion might have been detectable. In this paper, “ozone depletion” refers to a local decrease in ozone concentration at a given location or altitude attributable to ODS. Because chemical and dynamical processes influence ozone differently across vertical layers, their effects can mask one another when integrated into total column ozone, potentially obscuring early signals of change. While total column ozone has historically been a key policy-relevant indicator of ozone change, here we focus specifically on a thought experiment targeting altitude-resolved ozone change (consistent with what modern satellite observations provide) to identify when and where the first signals of ozone depletion could have been detectable.

Stratospheric ozone can be affected by changes in photochemistry driven by multiple factors. These include aerosol changes due to volcanic eruptions (15), changes in concentrations of CH_4_, N_2_O, and CFCs (16), temperature-dependent chemical processes (17), and stratospheric dynamics (9). The most commonly used CFCs, CFC-11, and CFC-12, were widely employed starting in the 1950s, with their concentrations increasing rapidly after about 1960 (18). However, another ODS, CCl_4_, had already been used as an industrial solvent in the United States as early as 1914, with widespread application by the 1930s (19)—roughly two decades before CFCs. Both firn air measurements (air of past atmospheres trapped in partially consolidated snow) and bottom–up inventory estimates (based on historical industrial production and usage records) indicate that CCl_4_ surface concentrations of 30 to 40 ppt were already present by 1950 (19, 20), a time when the total concentration of CFCs was still below 10 ppt (18, 20, 21).

In this thought experiment, we assume that the capability to monitor and simulate global ozone concentrations with state-of-the-art accuracy existed as early as 1950. Our experiment relies on modern large initial-condition ensembles, in which fully coupled chemistry-climate models are run many times from different atmospheric and oceanic initial conditions under identical external forcings. Each realization contains both natural internal variability and the responses to external forcings; hence, the ensembles allow these components to be clearly separated (14, 22–24) and support rigorous statistical analyses for detecting and attributing ozone depletion and recovery (12, 25). Here, detection refers to the anthropogenic signal being distinguishable with high statistical confidence from natural variability. This refers to the physical emergence of the ozone depletion signal and is independent of whether contemporaneous instruments could have measured it.

Few studies have reported ozone depletion prior to the 1980s (26), and no study has attempted to determine how early human-caused ozone depletion might have been detectable. Our thought experiment addresses multiple unexplored questions regarding when human fingerprints on the ozone layer might first have been detected, beginning with consideration of global observational coverage. Informed by that analysis, we also consider whether detection could have been achieved if observations had been available at specific sites only. We examine when three different layers (the upper, middle, and lower stratosphere, which are accessible with different observational methods) might have first displayed the onset of detectable changes, and how results for these individual layers compare to the results for global fingerprints across all three layers (accessible only by satellite). We will show that ozone depletion could have been detected with high confidence in the upper stratosphere before 1960, prior to widespread use of CFC-11 and CFC-12, and more than 20 y before the discovery of the Antarctic ozone hole.

## Results

### Ozone and ODS Evolutions.

**Fig. 1.**
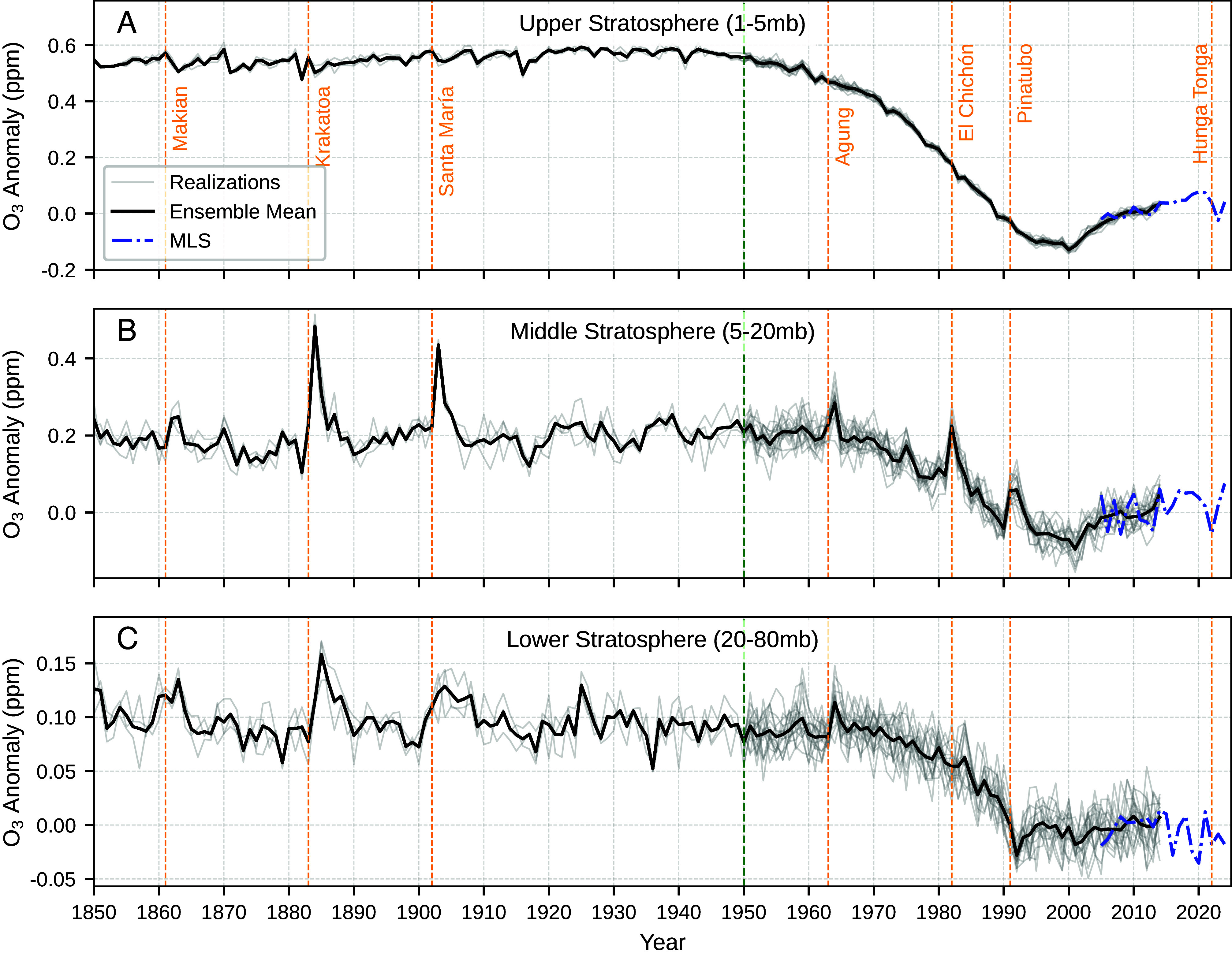
Observed and simulated evolution of global-mean stratospheric ozone after removal of the solar cycle. Annual-mean time series of global-mean, layer-average ozone volume mixing ratio in the (*A*) upper (1 to 5 mb), (*B*) middle (5 to 20 mb), and (*C*) lower stratosphere (20 to 80 mb), after removal of the solar cycle signal. Results are anomalies relative to averages over 2005–2014. Three WACCM realizations are available for the period 1850–1950 to characterize the ozone evolution during the preozone depletion era. Sixteen WACCM realizations are available for 1950–2014; a vertical green dashed line at 1950 marks the transition. Model ozone results are from simulations with combined forcing by ODS and GHG. Observations from the MLS during 2005–2024 are also shown, and the effect of the solar cycle on ozone has been removed using a standard regression method (*Methods*). The ozone evolution prior to solar cycle removal is shown in *SI Appendix*, Fig. S1. Individual ensemble members are shown in gray, and the ensemble-mean time series is in black. Major volcanic eruption dates are indicated by vertical orange lines; smaller eruptions are not shown. MLS ozone after solar cycle removal for 2005–2024 is shown as a dashed blue line.

We analyze simulated and observed annual-mean ozone evolution in the upper, middle, and lower stratosphere. The simulations are based on the Community Earth System Model–Whole Atmosphere Community Climate Model (CESM-WACCM6; hereafter referred to as WACCM) (27, 28). The WACCM initial-condition ensemble consists of 16 free-running, coupled ocean–atmosphere simulations for the period 1950–2014, driven by estimated historical variations in the solar forcing (29), volcanic eruptions (30), and concentrations of greenhouse gases (GHG) and ODS (18). Three of these members were initialized earlier, in 1850. Calculating global and layer averages reduces internal variability, allowing clear identification of ozone change in all three layers (see Fig. 1, which shows changes compared to a 2005–2014 average). The influence of the solar cycle on ozone has been removed to better display the anthropogenic impact (see also *SI Appendix*, Fig. S1 without solar cycle removal). The ozone mixing ratios observed by the Microwave Limb Sounder (MLS) are also shown in Fig. 1. For the period of overlap with MLS, both magnitude of the observed ozone changes and the interannual variability are closely captured by the WACCM ensemble. Furthermore, the latitude–altitude variability pattern in WACCM matches that in MLS (*SI Appendix*, Fig. S3). Together, these comparisons indicate that WACCM provides a realistic representation of the observed stratospheric ozone changes and variability.

Major volcanic eruptions sporadically increase or decrease global ozone levels for a few years in both the middle and lower stratosphere. Increases are primarily due to heterogeneous reactions on the volcanic aerosols, which reduce NOx levels and subsequently suppress ozone loss via the NOx cycle (31). After the buildup of ODS in the latter part of the 20th century, such eruptions caused ozone decreases in the lower stratosphere [see, e.g., the response to Pinatubo in the early 1990s (15, 32)].

**Fig. 2.**
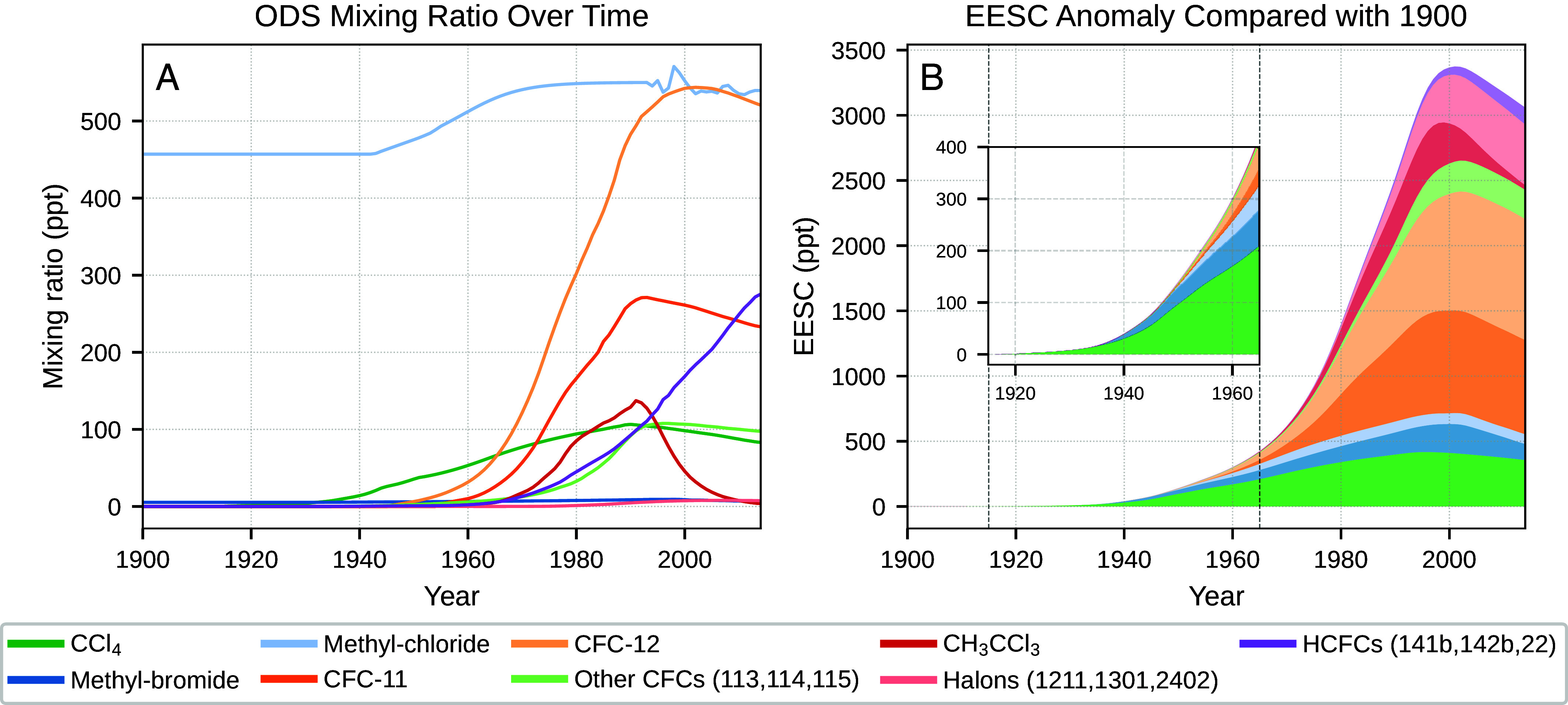
Evolution of global-mean surface ODS mixing ratios and upper stratospheric EESC. (*A*) Global-mean annual-mean surface mixing ratios of ODS, derived from observations and used as input to the WACCM simulations. For clarity, halons, HCFCs, and CFC-113, -114, and -115 are grouped together in the plotted time series. (*B*) Equivalent effective stratospheric chlorine (EESC) in air with a mean age of 5.5 y, calculated from the ODS mixing ratios shown in panel *A*. This age corresponds approximately to the upper stratosphere at midlatitudes. The EESC anomaly is shown relative to 1900 to highlight the anthropogenic contribution. A zoomed-in view of 1915–1965 highlights the early contribution of ODS to EESC.

Overall, upper stratospheric ozone features minimal internal variability and strong sensitivity to ODS, making it an optimal region for detecting anthropogenic influences on ozone (11, 33–35), which begin to emerge in the late 1950s (Fig. 1). This early ozone depletion is driven by early ODS emission. Ozone-depleting substances are transported up to the stratosphere, where they undergo photolysis to release inorganic halogens that catalyze ozone destruction (6). Surface concentrations of ODS, derived from firn air and direct atmospheric measurements (18), are shown as global and annual means in Fig. 2*A* and are used as model forcings.

Equivalent effective stratospheric chlorine (EESC) has been used to assess the collective impact of chlorine and bromine released from ODS on stratospheric ozone (36, 37). Prior to 1920, stratospheric halogens were predominantly of natural origin, primarily from emissions of methyl chloride and methyl bromide (18). Here, we focus on EESC anomalies relative to year 1900 levels, representing the anthropogenic contribution to EESC, in air with a mean age of 5.5 y—corresponding roughly to the upper stratosphere at midlatitudes (Fig. 2*B*). During 1920–1960, CCl_4_ was the dominant contributor to anthropogenic EESC, accounting for ~69% in 1950 and ~56% in 1960. The large increase in CCl_4_ from 1920–1960 was mainly due to its widespread use as an industrial solvent (38).

The combined effects of GHG alone (CO_2_, CH_4_, and N_2_O) would have caused positive ozone trends in the middle and upper stratosphere (*SI Appendix*, Fig. S2). These GHG-induced trends are much smaller than the net negative trends in ozone in all three layers in the ODS+GHG simulations. By partially offsetting ozone loss, rising GHG concentrations moderated ozone depletion and delayed its early detection. Interestingly, the ozone response to the Pinatubo eruption in the lower stratosphere is opposite under high and low ODS conditions (*SI Appendix*, Fig. S2). This is mainly attributed to different ClOx cycle intensities in these two sets of simulations, highlighting the importance of chlorine loading on the volcanically driven ozone effects (15, 32).

### Ozone Trend and Internal Variability.

**Fig. 3.**
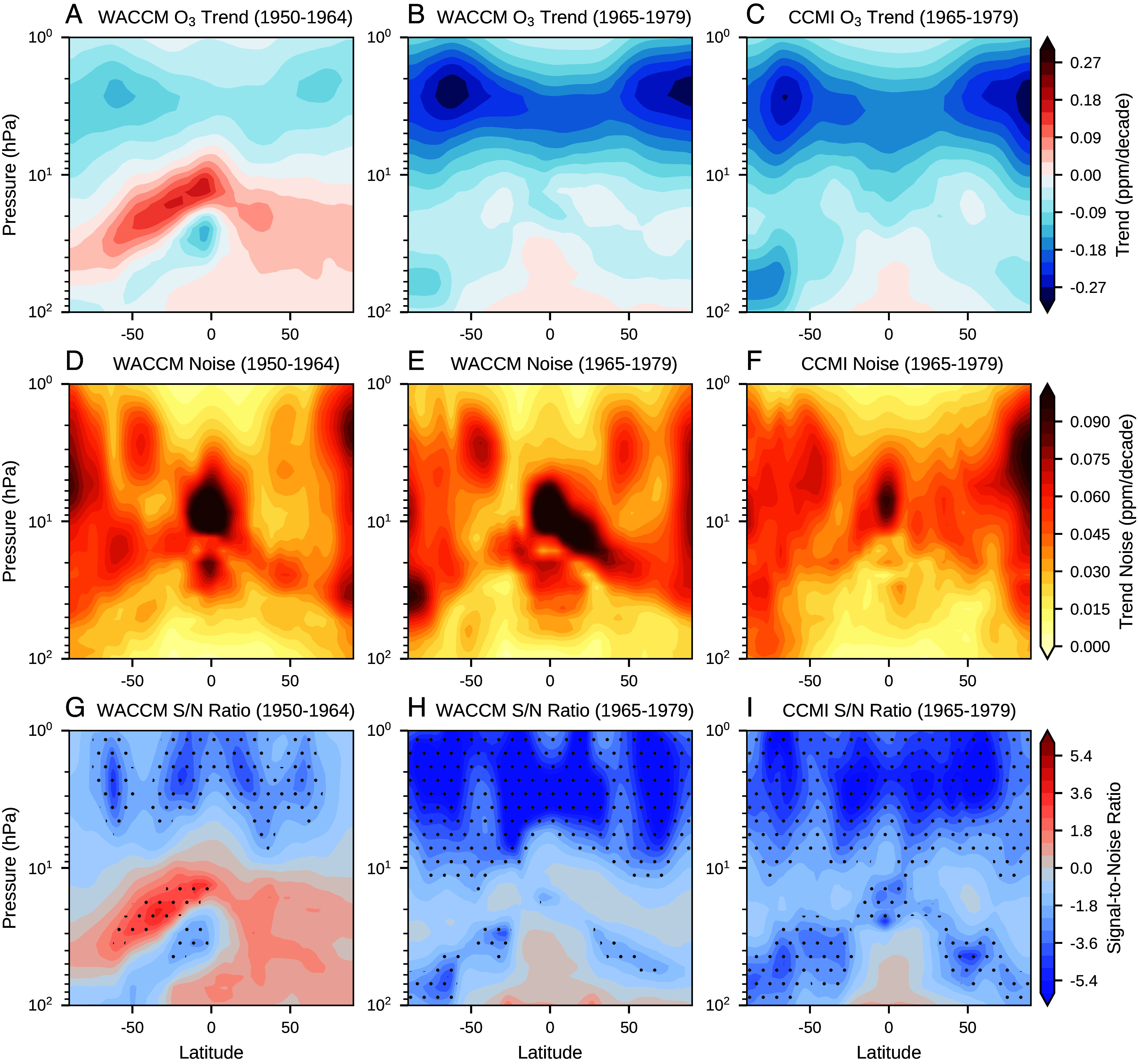
Spatial patterns of 15-y ozone trends, internal variability, and signal-to-noise ratios during different ozone depletion periods from WACCM and CCMI. (*A*–*C*) Ensemble-mean ozone trends across all model ensemble members over selected 15-y periods: (*A*) 1950–1964 (WACCM), (*B*) 1965–1979 (WACCM), and (*C*) 1965–1979 (CCMI). (*D*–*F*) Internal variability (noise) of the corresponding 15-y trends for the same time periods and models. (*G*–*I*) Signal-to-noise (S/N) ratios, calculated by dividing each signal in the *Top* row by its corresponding noise estimate in the *Middle* row. Stippling indicates regions where the S/N ratio exceeds 1.96, corresponding to the 95% confidence level.

We next analyze the simulated ensemble-mean spatial pattern of ozone trends (“signal”), the internal variability of trends (“noise”), and the signal-to-noise ratio (S/N) during selected early depletion periods (Fig. 3). As examples, we first present 15-y trends calculated over two periods: 1950–1964 and 1965–1979. Results for different trend lengths are discussed below. During the period 1950–1964, negative ozone trends were already widespread in the upper stratosphere (Fig. 3*A*), driven by increasing EESC. The spatial patterns of trends in the middle and lower stratosphere—ozone increases near the mid-stratosphere and decreases in the tropics near 30 mb—are due to the 1963 Agung eruption.

Fig. 3 *B* and *C* show the ozone trends over 1965–1979 from both WACCM and the latest Chemistry–Climate Model Initiative 2022 (CCMI-2022) (39), which includes 19 realizations across nine models. The CCMI simulations do not begin until 1960, so the 1950–1964 trend is unavailable, limiting early ozone detection using that ensemble. Unlike the single-model WACCM ensemble, the CCMI multimodel ensemble includes intermodel differences in both forced responses and internal variability (23). Despite this difference in the two ensembles, the general agreement between the WACCM and CCMI results in Fig. 3 supports the robustness of these spatial patterns across models.

Over 1965–1979, the ozone decline intensified in the upper stratosphere due to continued EESC increases. The strongest ozone depletion is found in the upper stratosphere over mid-latitude to polar regions, primarily associated with enhanced ClO concentrations arising from lower methane abundances in descending air, which affects the partitioning of inorganic chlorine (40, 41).

In order to assess signal detection, both the trend and the internal variability are important. Fig. 3 *D* and *E* show the spatial patterns of internal variability estimated from WACCM, defined as the SD of 15-y ozone trends across ensemble members after removing the mean forced response. The largest noise values are at the tropical 10 mb level, associated with the Quasi-Biennial Oscillation (QBO) (42, 43). Fig. 3*F* shows the CCMI internal variability pattern, computed after excluding the highest and lowest trend values to reduce bias from outlier models. CCMI and WACCM internal variability patterns are broadly consistent, although CCMI shows weaker variability in the QBO region. This discrepancy likely arises because not all CCMI models simulate an internally generated QBO, reducing QBO-related variability (39, 44). Notably, both the WACCM and CCMI ensembles exhibit minimal variability in the tropical upper stratosphere, more specifically near 20°S and 20°N, resulting in the largest S/N in this region (Fig. 3 *G*–*I*). Regions where S/N exceeds the 95% confidence threshold are stippled. Importantly, statistically significant ozone depletion was detectable as early as 1950–1964, becoming widespread throughout the upper stratosphere during 1965–1979.

To assess the realism of the modeled variability, especially in the key region of the upper stratosphere, we compared the modeled ozone internal variability with MLS (45) observations for the period 2005–2014 in *SI Appendix*, Fig. S3. The spatial patterns are broadly consistent, although MLS exhibits stronger variability in the QBO region. A comparison of WACCM and MLS variability in the upper stratosphere shows close agreement in both the normalized variability (relative to their respective climatologies) and in the absolute ozone mixing ratios (*SI Appendix*, Fig. S3 *C* and *D*).

### Detection of Early Ozone Depletion.

**Fig. 4.**
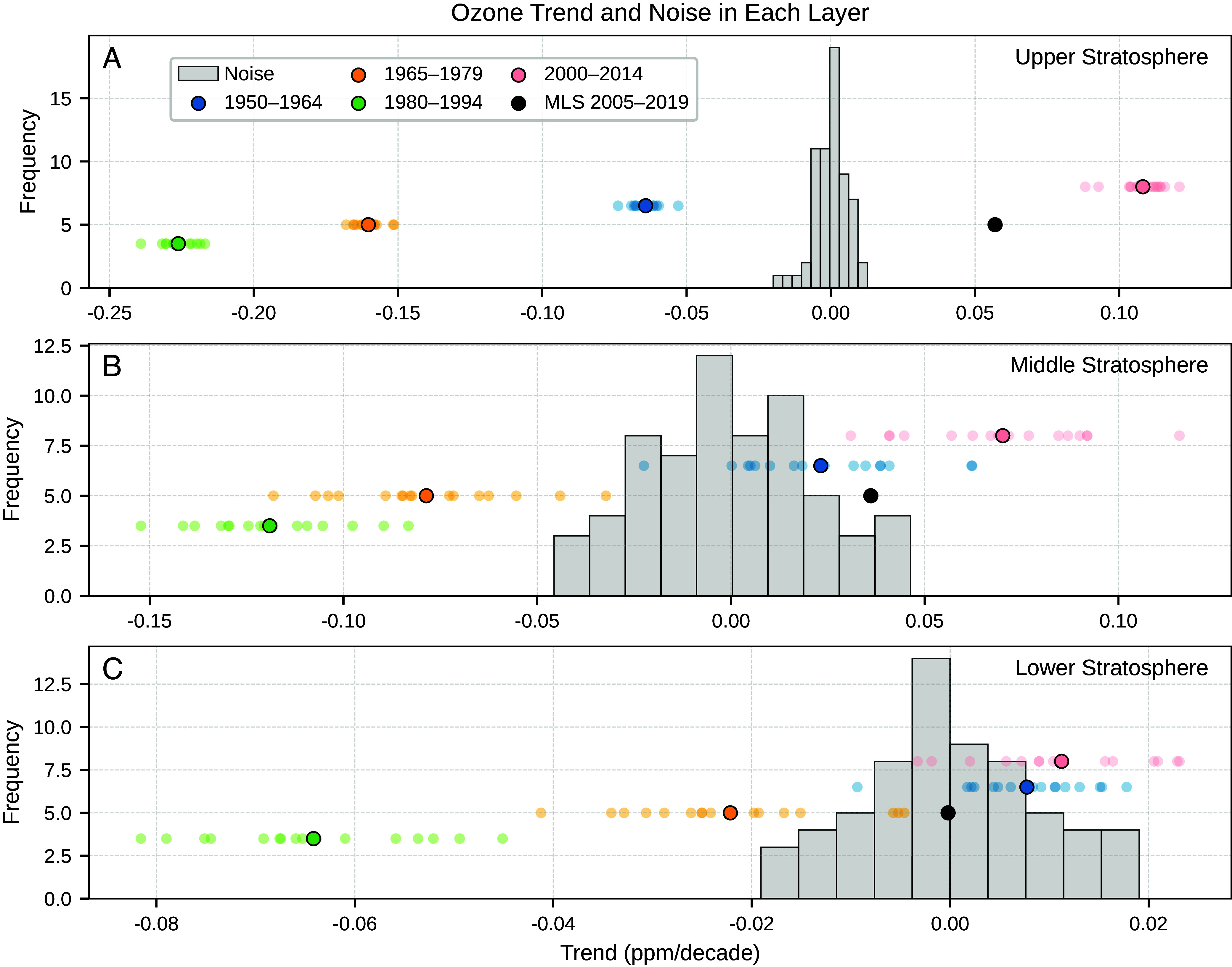
Layer-mean 15-y global ozone trends and internal variability during different ozone depletion periods in different stratospheric layers. Fifteen-year layer-mean ozone trends and their internal variability (noise) in the (*A*) upper, (*B*) middle, and (*C*) lower stratosphere. Trends and variability are calculated from the ozone time series shown in Fig. 1. Ozone trends for 1950–1964, 1965–1979, 1980–1994, and 2000–2014 from WACCM are shown in lighter circles for individual realizations and darker circles with black outlines for the ensemble mean. Observed trends from MLS (2005–2019) are shown as black points. Note the different time periods between simulated and observed ozone recovery periods due to the limited observational record for MLS. Histograms represent the simulated internal variability in the 15-y trends of residuals (defined as the difference between each individual model realization and the ensemble-mean ozone time series) during the four periods. The sample size in each histogram is 64 (i.e., there are four analysis periods, each with 16 sets of residual 15-y trends).

In Fig. 4, we analyze 15-y ozone trends and compare them with internal variability across different periods and stratospheric layers. The analysis is based on the global-mean layer-average data in Fig. 1. In the upper stratosphere (Fig. 4*A*), the trend for 1950–1964 is well beyond the range of WACCM internal variability, with an S/N ratio of approximately 11. In this layer, therefore, global-mean ozone depletion is already statistically detectable in the first 15 y following 1950. The trend becomes increasingly negative in the subsequent periods of 1965–1979 and 1980–1994, reflecting the continued rise in ODS. In contrast, the trend reversed sign in 2000–2014, with a large positive S/N of 18, indicating statistically significant ozone recovery during this period (46). Observations from MLS for 2005–2019 also show a clear recovery in the upper stratosphere, with an S/N of 10 (note the different analysis time periods in MLS and the models due to the limited observational record and the ending of the simulations in 2014).

In the middle and lower stratosphere (Fig. 4 *B* and *C*), ozone shows no significant depletion in the early 1950–1964 period. However, significant negative trends in both regions emerged during 1965–1979 and became more pronounced in 1980–1994. WACCM simulations indicate that ozone in the middle stratosphere recovers significantly (at the 95% confidence level) during 2000–2014, while the MLS trend over 2005–2019 also shows recovery, though only at the 90% confidence level. In the lower stratosphere, however, neither WACCM nor MLS shows statistically significant evidence of global-mean ozone recovery.

**Fig. 5.**
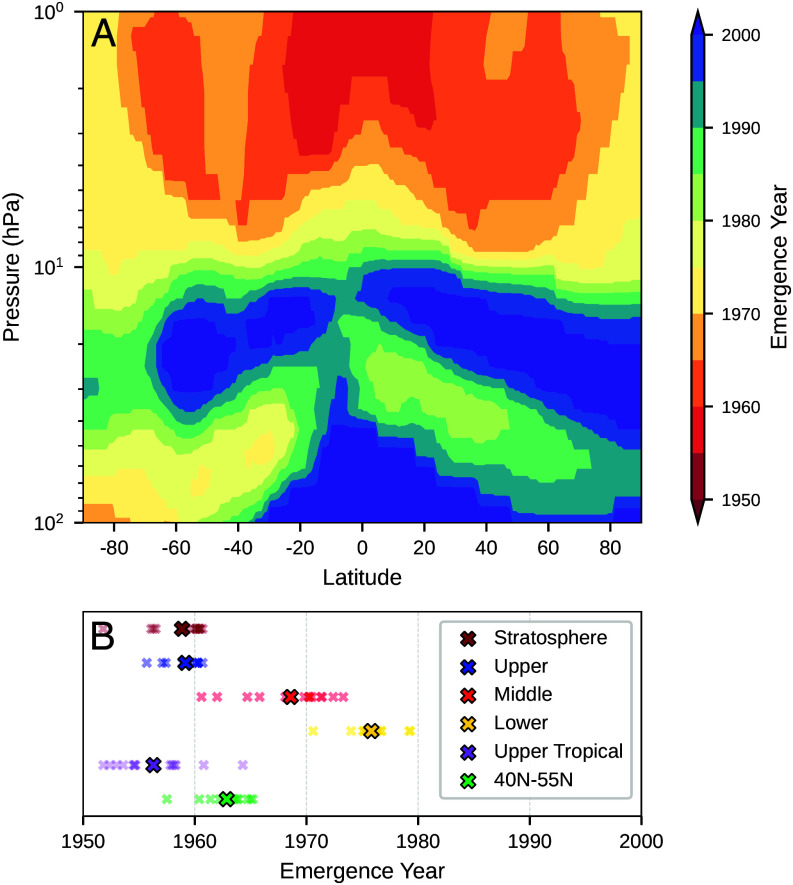
Emergence time of ozone depletion in different domains. (*A*) Spatial pattern of the WACCM-derived emergence time based on local signal-to-noise (S/N) analysis of “solar cycle removed” ozone results. The signal is defined as the linear trend starting in 1950 and ending incrementally from 1951–2000. The noise is the internal variability of the trend over the corresponding periods. Emergence time is defined as the first year in which the S/N ratio exceeds the 95% confidence threshold and remains at or above it through the end of the detection window (1950–2000). (*B*) Emergence times estimated for different spatial domains using a pattern-based fingerprinting method. Lighter circles represent individual realizations; darker circles with black outlines denote ensemble means. Six domains are included: the entire stratosphere (90°S–90°N, 1 to 80 mb), upper stratosphere (90°S–90°N, 1 to 5 mb), middle stratosphere (90°S–90°N, 5 to 20 mb), lower stratosphere (90°S–90°N, 20 to 80 mb), upper tropical stratosphere (30°S–30°N, 1 to 5 mb), and northern mid-latitudes (40°N–55°N, 1 to 80 mb).

We next assess the timing and spatial pattern of ozone depletion emergence using a rigorous S/N analysis. This assessment is conducted both for individual zonal means (“local” analysis) and for the latitudinal spatial pattern. Fig. 5*A* shows the year of emergence based on the local S/N. Emergence time is defined as the first year the S/N exceeds the 95% confidence threshold and remains above it throughout the depletion period (1950–2000). If monitoring had for example begun in 1950, the earliest emergence at 95% confidence occurs in 1957 in the tropical upper stratosphere. By 1974, when Molina and Rowland first hypothesized ozone depletion due to CFCs (3), significant depletion may already have been detectable across nearly the entire upper stratosphere. We find that between 1950 and 1960, the model-calculated internal variability of column ozone is at least two times greater than the simulated column depletion at all latitudes; the depletion is therefore not detectable based on the local analysis. This result highlights the key information provided by the vertical profile. In the lower stratosphere over Antarctica, the ozone depletion in WACCM became detectable in 1976, approximately a decade before the observations of depletion by Farman et al. (1).

Our pattern-based fingerprint method for identifying the emergence of ozone depletion (14, 47) is applied to individual stratospheric layers and to the full stratosphere (*Methods*). For each of our three layers (and when all three layers are considered jointly), we use the leading Empirical Orthogonal Function (EOF) of ensemble-mean zonal-mean ozone change as the fingerprint pattern (*SI Appendix*, Fig. S4). Starting from 1950, we computed the element-wise covariance between this fingerprint and the ozone trend pattern in individual realizations (the “signal”), as well as the covariance between the fingerprint and internal variability (“noise”), providing a measure of signal and noise spatial similarity for different trend lengths (*SI Appendix*, Fig. S5). A key feature of this method is that signal and noise are evaluated on the same time scales as the analysis period increases. The signal tends to increase with longer trend lengths, while the noise decreases, leading to larger S/N.

Results of the pattern-based S/N analysis are shown in Fig. 5*B*. The ensemble-mean emergence times are approximately 1959 for the entire stratospheric latitude-altitude domain, and 1959, 1968, and 1976 for the upper, middle, and lower stratosphere, respectively. To assess the detectability of ozone depletion in scenarios where accurate global measurements might be unavailable at the earliest times, we repeated the emergence analysis using spatially limited domains. In the tropical upper stratosphere, where the earliest emergence is observed, the ensemble-mean emergence time is around 1957, consistent with the local S/N analysis. In the Northern Hemisphere mid-latitudes, where the first stratospheric ozone measurements were made (48), depletion becomes detectable around 1963, a few years later than in the global upper stratosphere.

In the results reported above, we isolated anthropogenic ozone signals by removing the influence of the solar cycle. Given its large impact on stratospheric ozone (*SI Appendix*, Fig. S1), we also examine S/N results without solar cycle removal. The roughly 11-y solar cycle primarily affects early detection, with minimal influence on later emergence (*SI Appendix*, Fig. S5). This limited effect reflects i) the smaller magnitude of solar-driven ozone changes compared to ODS impacts at high ODS concentrations, and ii) the diminishing influence of periodic variability over longer trend lengths. In the local S/N analysis, the earliest emergence without solar cycle removal is delayed to 1963 (*SI Appendix*, Fig. S6). Pattern-based detection shows similar delays, with signal emergence shifting to about 1962 in the entire stratosphere and upper stratosphere, and to 1963 in the tropical upper stratosphere. For other regions, including the solar cycle causes only minor (1 to 2 y) or negligible delays. Detection results for S/N analyses with and without QBO removal yield minor or negligible changes (*SI Appendix*, Fig. S7).

### Summary and Outlook.

We have revisited the timeline of ozone depletion using a “thought experiment” to explore when and where ODS-induced changes to the ozone layer could have first been detectable. Leveraging ensembles of well-validated chemistry-climate models, and assuming the availability of state-of-the-art global ozone monitoring from 1950 onward, we find that the earliest ozone depletion emergence appears in the tropical upper stratosphere as early as 1957—more than 15 y prior to the seminal work by Molina and Rowland (3).

In addition, we found that under varying stratospheric chlorine loadings, volcanic eruptions have complex impacts on ozone. For example, the 1991 Pinatubo eruption caused a decrease in lower stratospheric ozone under current high-ODS conditions but would increase ozone in a low-ODS atmosphere (*SI Appendix*, Fig. S2*C*). The most commonly used method in ozone studies, multiple linear regression (49), models the volcanic effect as linearly proportional to aerosol optical depth. While this approximation holds under present high-ODS conditions, caution is warranted when applying it to earlier volcanic events or future scenarios with declining ODS concentrations.

Our results highlight the critical importance of a thorough understanding of internal variability. The earliest emergence found does not occur in regions with the strongest negative ozone trends, but rather in areas of low ozone variability. Additionally, both local and pattern-based S/N analyses highlight the value of global, height-resolved observations spanning extended periods, raising concerns about the impending gap in satellite-based stratospheric measurements (50, 51).

Our thought experiment also suggests that ODS have impacted the stratospheric ozone layer for nearly 70 y, since the late 1950s, first initiated by human use of CCl_4_ as a solvent. This was more than 20 y before the first regulatory action targeting a different set of chemicals, namely national bans on CFC-11 and CFC-12 in spray cans, and almost 30 y before the discovery of the Antarctic ozone hole. We cannot know whether or not knowledge of such early ozone depletion would have affected the evolution of human actions regarding the use of halocarbons.

## Methods

### Model Simulations.

We employ the fully coupled CESM2-WACCM6 (27, 28) to quantify forced responses in ozone and to estimate internal variability. WACCM6 includes fully coupled ocean–atmosphere processes and a well-validated interactive QBO, a major source of stratospheric internal variability. It also features interactive atmospheric chemistry, incorporating a comprehensive set of gas-phase and heterogeneous reactions, including the Ox, NOx, HOx, ClOx, and BrOx chemical families. These features allow WACCM to realistically capture both ozone depletion and stratospheric internal variability.

The model is driven by historical forcings consistent with the Coupled Model Intercomparison Project Phase 6 (CMIP6) (30), including the solar cycle (29), volcanic aerosols (30), GHG, and ODS (18). A three-member ensemble of historical simulations covering 1850–2014 is available as part of CMIP6. An additional thirteen-member ensemble started on January 1, 1950, using initial conditions perturbed slightly from this three-member ensemble (52). These 13 simulations span the years 1950–2014; each is driven by the same historical forcings. This yields a three-member ensemble for the period 1850–1950, and a 16-member ensemble for 1950–2014, the period of primary interest for our detection study. This large ensemble size enables robust estimation of both the forced response and internal variability.

EESC is calculated based on the historical ODS forcing used in the model, following the method from the 2022 World Meteorological Organization (WMO) ozone assessment (6, 37).

We also present results for a “GHG-only” scenario using a four-member ensemble under low ODS conditions, similarly initialized with different perturbations to atmospheric initial conditions in 1950. This scenario is forced identically to the historical simulations, except that ODS concentrations are fixed at 1950 levels. Although this “low ODS state” includes a very small anthropogenic ODS contribution (as shown in EESC results for 1950 in Fig. 2*B*), the 1950 concentrations are negligible compared to later ODS levels.

The CCMI-2022 simulations (39) rely on a suite of state-of-the-art chemistry–climate models. We use the free-running REF-D2 simulations, which span 1960–2100 and follow the WMO baseline scenario (53) for ODS and the SSP2-4.5 scenario from CMIP6 (54). We focus on initial-condition ensembles and exclude ensembles that differ only by model parameters or forcings within the same model (e.g., CNRM-MOCAGE). In total, we include 19 available realizations across nine different models. Some CCMI-2022 models do not include interactive ocean components, and some use nudged rather than interactive QBOs (39, 44). These differences can introduce biases and uncertainties in the representation of internal variability (23).

### Satellite Data.

The Microwave Limb Sounder (MLS) has provided well-validated ozone observations from August 2004 to the present (55, 56). We use version 5 of the MLS Level 3 monthly mean ozone mixing ratios on pressure coordinates. The Level 3 product spans latitudes from 82° S to 82° N, with a 4° latitude resolution. The high spatial coverage enables accurate calculation of global-mean ozone concentrations, which are weighted by the cosine of latitude to account for decreasing surface area toward the poles. Annual means from 2005–2024 are used in this study.

Ozone variability from MLS is estimated using a standard regression-based approach (57) applied to the overlap period of 2005–2014 (when both MLS observations and WACCM output are available). At each grid point (defined by latitude and pressure level),

$${\mathrm{Ozone}}_{\mathrm{MLS}}=a\times {\mathrm{Ozone}}_{\mathrm{ensemble}-\mathrm{mean}}+\varepsilon ,$$

where ${\mathrm{Ozone}}_{\mathrm{MLS}}$ is the annual mean ozone concentration time series from MLS in 2005–2014, and ${\mathrm{Ozone}}_{\mathrm{ensemble}-\mathrm{mean}}$ is the ensemble-mean of the 16-member WACCM ozone time series over the same period, representing the forced response. The coefficient a is a scaling parameter that minimizes the difference between MLS observations and the ensemble mean. The residual term ε is the component of ${\mathrm{Ozone}}_{\mathrm{MLS}}$ not explained by the forced response, and the SD of the 10 residual annual-mean values at each grid point (ε) is the observed residual variability in ozone. Note that, due to the limited length of the observational record and the end of the WACCM simulations in 2014, we are constrained to compare WACCM and observed variability of annual-mean ozone concentrations over 2005–2014 (*SI Appendix*, Fig. S3). The short MLS data cannot be used to estimate observed variability in ozone trends on the longer (15-y) timescales of interest here. We therefore rely on WACCM and CCMI results for estimating longer-timescale variability.

### Signal and Noise Definition and Analysis.

For WACCM, the pattern of the signal (forced response) is calculated as the ordinary least-squares linear trend in ensemble-mean ozone at each latitude and pressure level. To estimate internal variability, we first subtract the ensemble-mean ozone time series from each individual model realization to isolate the residuals. The internal variability is the SD of the linear trends of these residuals across all realizations.

For the CCMI simulations, we use 19 realizations from nine different models, with an uneven number of realizations across models. To avoid overrepresenting models with more realizations, we apply a weighting scheme where each model is given equal weight (i.e., one point per model) (14). This weight is evenly distributed among all realizations from that model. In this way, we include all available realizations while ensuring that each model contributes equally to the overall ensemble mean. Since multimodel ensembles include substantial cross-model differences that can inflate the estimated internal variability (23), we reduce the influence of outliers by excluding the highest and lowest CCMI results at each grid point and then calculating internal variability.

For the analysis of local emergence, we calculate the signal and noise of ozone trends over periods starting in 1950 and ending incrementally from 1951–2000. The emergence time is defined as the first year when the S/N exceeds the 95% confidence threshold (1.96) and remains at or above this threshold for all longer analysis periods, until the end of the depletion period in 2000.

For the analysis of pattern-based emergence, we use a standard “fingerprint” method (14, 47). The fingerprint pattern is defined as the leading EOF of ensemble-mean zonal-mean ozone change in each domain (upper, middle, and lower stratosphere) during the period 1950–2014 (*SI Appendix*, Fig. S4). We search for these fingerprints in each of the 16 WACCM historical realizations. For each trend period starting in 1950 and ending incrementally from 1951–2000, we compute the ozone trend patterns and the residual trend patterns from all 16 realizations. We then calculate the element-wise uncentered spatial covariance between the fingerprint and the trend pattern in each of the 16 WACCM realizations. Simultaneously, we calculate the element-wise uncentered spatial covariances between the fingerprint and each individual residual trend pattern. The SD of these covariances represents the internal variability (noise). This procedure yields an S/N ratio for each trend length. The emergence time is defined as the first year when S/N exceeds the 95% confidence threshold (1.96) and then remains at or above it through the end of the period (*SI Appendix*, Fig. S5), consistent with the definition used in the local emergence analysis. We apply this fingerprint analysis to several different regions, defined as follows: stratosphere (90°S–90°N; 1-80 mb), upper stratosphere (90°S–90°N; 1 to 5 mb), middle stratosphere (90°S–90°N; 5 to 20 mb), lower stratosphere (90°S–90°N; 20 to 80 mb), upper tropical stratosphere (30°S-30°N; 1 to 5 mb), and Northern Hemisphere mid-latitudes (40°N-55°N;1 to 80 mb).

### Solar Cycle Removal.

The influence of the solar cycle on ozone has been removed using a standard regression-based method (49, 58). The solar cycle is a natural external forcing, and the ensemble-mean ozone concentration from WACCM contains this naturally forced response, as well as the ozone responses to anthropogenic ODS and GHG forcing. Ozone evolution can be broadly divided into three phases: a relatively stable period from 1850–1950, ozone depletion from 1950–1990 (or to 2000, depending on the region), and ozone recovery beginning after 2000. Because the three-member ensemble for 1850–1950 is too small to robustly distinguish the forced response from internal variability, and the recovery period after 2000 is too short for reliable trend analysis, we focus on the depletion period (1950–1990) to estimate the solar cycle’s effect. This time frame also avoids the strong perturbation from the 1991 Pinatubo eruption. At each grid point (defined by latitude and pressure level), we calculate the linear relationship between detrended ozone concentrations and the annual-mean total solar irradiance (TSI). This regression is then applied to the full period 1850–2014. This method effectively removes the solar signal, as shown in Fig. 1. To validate the removal, we check the correlation between the adjusted ozone time series and TSI across all three time periods; the correlations are all nearly zero, confirming that the solar cycle signal has been successfully removed. For the MLS ozone data, the solar cycle effect is similarly removed by regression with observed TSI from NOAA (https://www.ncei.noaa.gov/data/total-solar-irradiance/access/monthly/, last accessed: June 26, 2025), but applied to the 2005–2024 period.

### QBO Removal.

The influence of the QBO on ozone has been removed using a regression-based method (33). WACCM includes an internally generated QBO, so each realization exhibits a different QBO phase, and the ensemble mean smooths out this variability. As a result, the QBO only contributes to internal variability in our analysis. To remove its effect, ozone anomalies in each realization are regressed against tropical (5°S–5°N) zonal winds at 10 mb and 30 mb.

## Supplementary Material

Appendix 01 (PDF)

## Data Availability

Model output data and analysis code data have been deposited in Zenodo (59).
