# Supplementary material for: The emergence of human influence on the ozone layer by the 1960s
Source: Proc Natl Acad Sci U S A. 2026 Jun 29;123(28):e2608286123. doi: 10.1073/pnas.2608286123 (PMC13367830; doi:10.1073/pnas.2608286123)
Supplement: Supplementary file 1 — Appendix 01 (PDF) [file pnas.2608286123.sapp.pdf]

PNAS

Supporting Information for

The Emergence of Human Influence on the Ozone Layer by the 1960s

Jian Guan[1,*], Benjamin D. Santer[2], Peidong Wang[1], Qiang Fu[3], Rolando R. Garcia[4], Yaowei Li[1], Kane Stone[1], Douglas E. Kinnison[4], Jun Zhang[4], Gabriel Chiodo[5], Jean-Francois Lamarque[4,†], Susan Solomon[1,*]

1. Department of Earth, Atmospheric, and Planetary Sciences, Massachusetts Institute of Technology, Cambridge, MA, USA
2. School of Environmental Sciences, University of East Anglia, Norwich, UK
3. Department of Atmospheric and Climate Science, University of Washington, Seattle, WA, USA
4. National Center for Atmospheric Research, Boulder, CO, USA
5. Instituto de Geociencias, Consejo Superior de Investigaciones Cientificas, Madrid, Spain

* Corresponding author: Jian Guan; Susan Solomon

[†] now at Climate Modeling and Analysis LLC, Superior, CO

Email: jianguan@mit.edu; solos@mit.edu

This PDF file includes:

Supplement Figures 1 to 7

Figure S1. Observed and simulated evolution of global-mean stratospheric ozone before removal of the solar cycle signal.

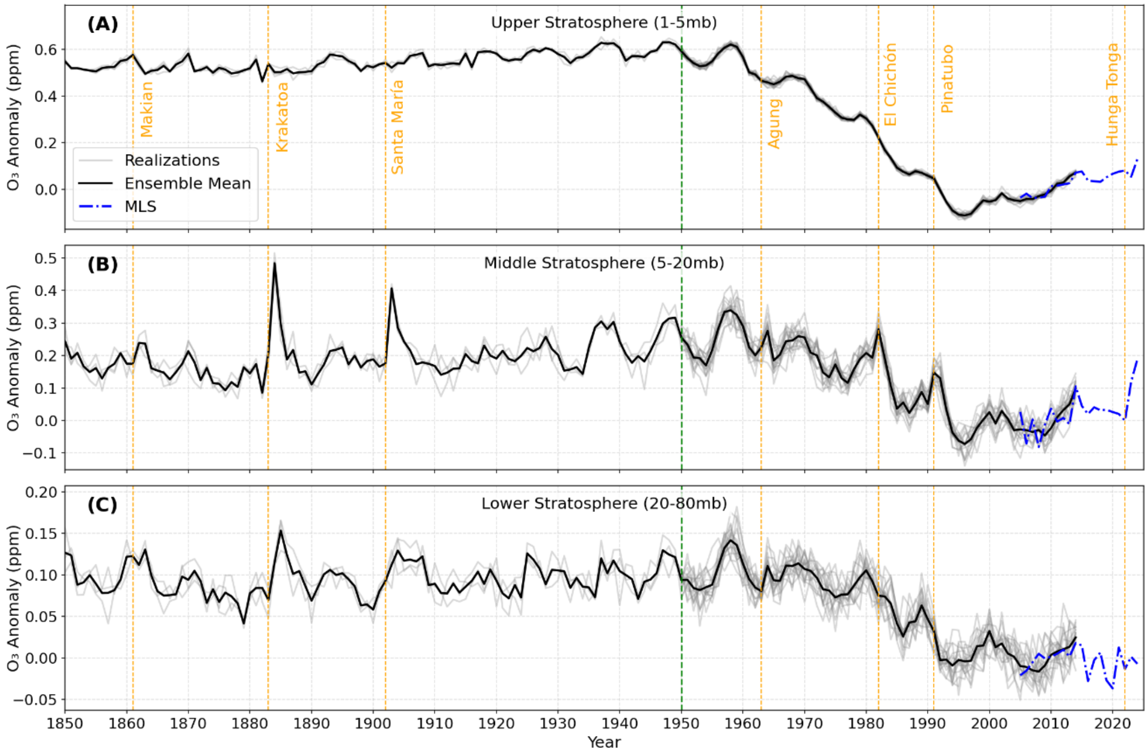

As for Fig. 1, but before removal of the solar cycle signal.

Figure S2. Evolution of global-mean stratospheric ozone after removal of the solar cycle signal in historical and GHG-only simulations.

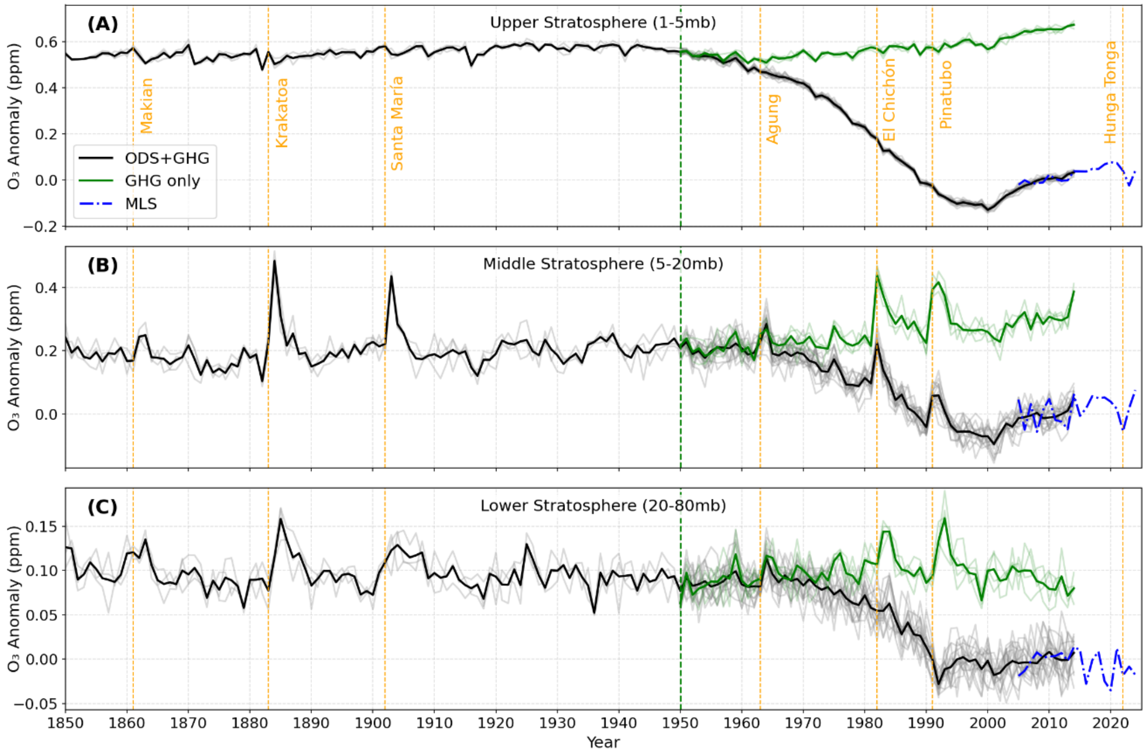

As for Fig. 1, but with the addition of GHG-only simulations. Four individual GHG-only realizations are shown as lighter green lines, and the ensemble mean is shown as a solid green line.

Figure S3. Observed and simulated variability of ozone mixing ratios during 2005–2014.

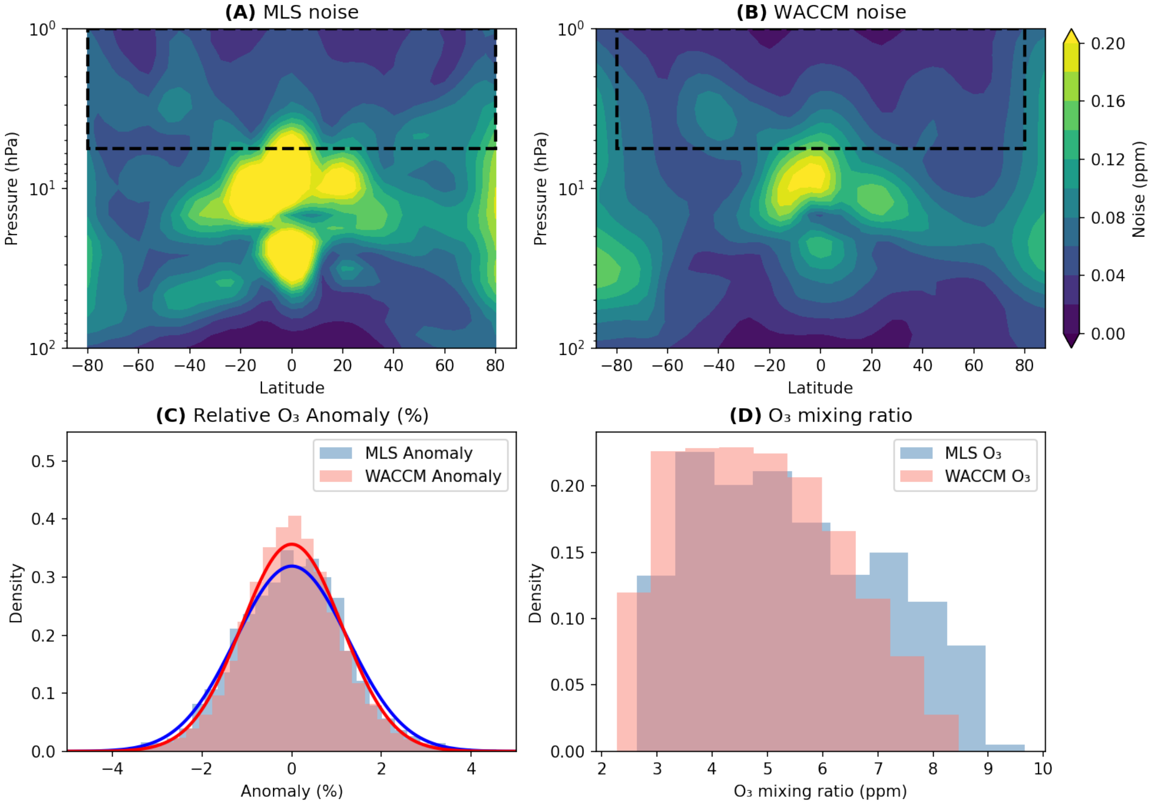

(A) Spatial pattern of ozone variability observed by MLS during 2005–2014, expressed as the standard deviation of residuals (defined as the difference between MLS measurements and the fitted WACCM ensemble-mean ozone time series). MLS covers 82° S to 82° N; regions outside this range are left blank.
(B) Corresponding pattern of ozone variability simulated by WACCM, defined as the standard deviation of residuals between each individual model realization and the ensemble mean.
(C) Relative variability of ozone residuals in MLS and WACCM, expressed as a percentage of the 2005–2014 climatological mean ozone mixing ratios in the upper stratosphere (boxed region).
(D) Absolute annual-mean ozone mixing ratios observed by MLS and simulated by WACCM during 2005–2014 in the upper stratosphere (boxed region).

Figure S4. Pattern of the ozone fingerprint

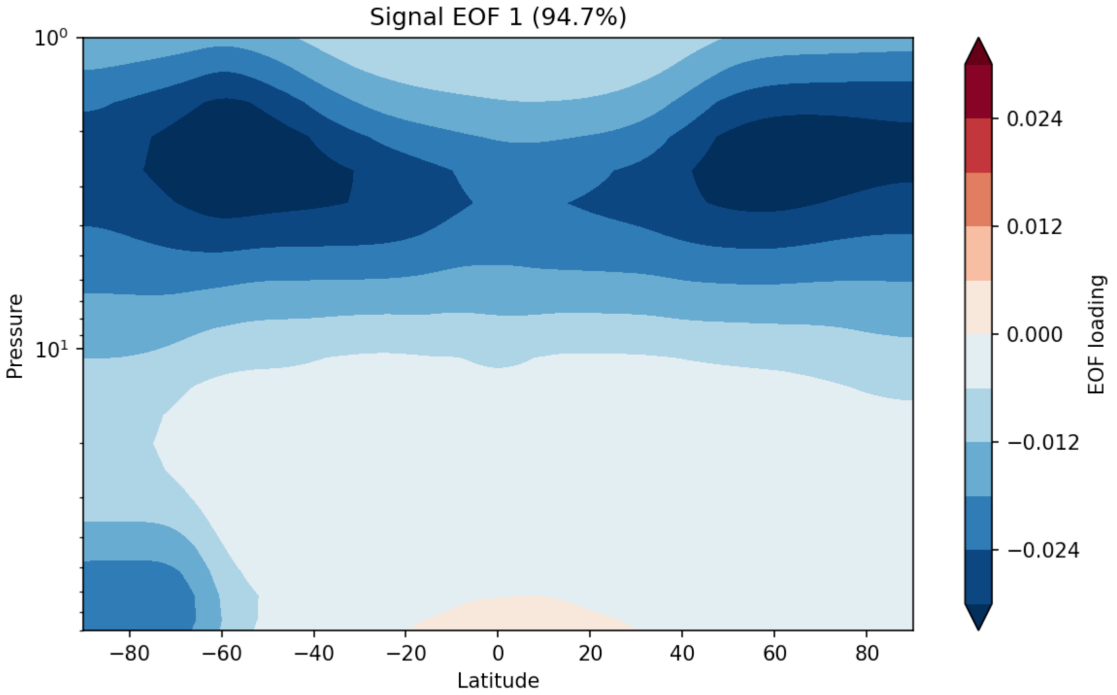

The fingerprint pattern is defined as the leading empirical orthogonal function (EOF) of ensemble-mean ozone change over a global domain during 1950–2014. This pattern explains 94.7% of the total variance.

Figure S5. Signal, noise, and signal-to-noise ratio from fingerprint analysis and sensitivity to solar cycle removal.

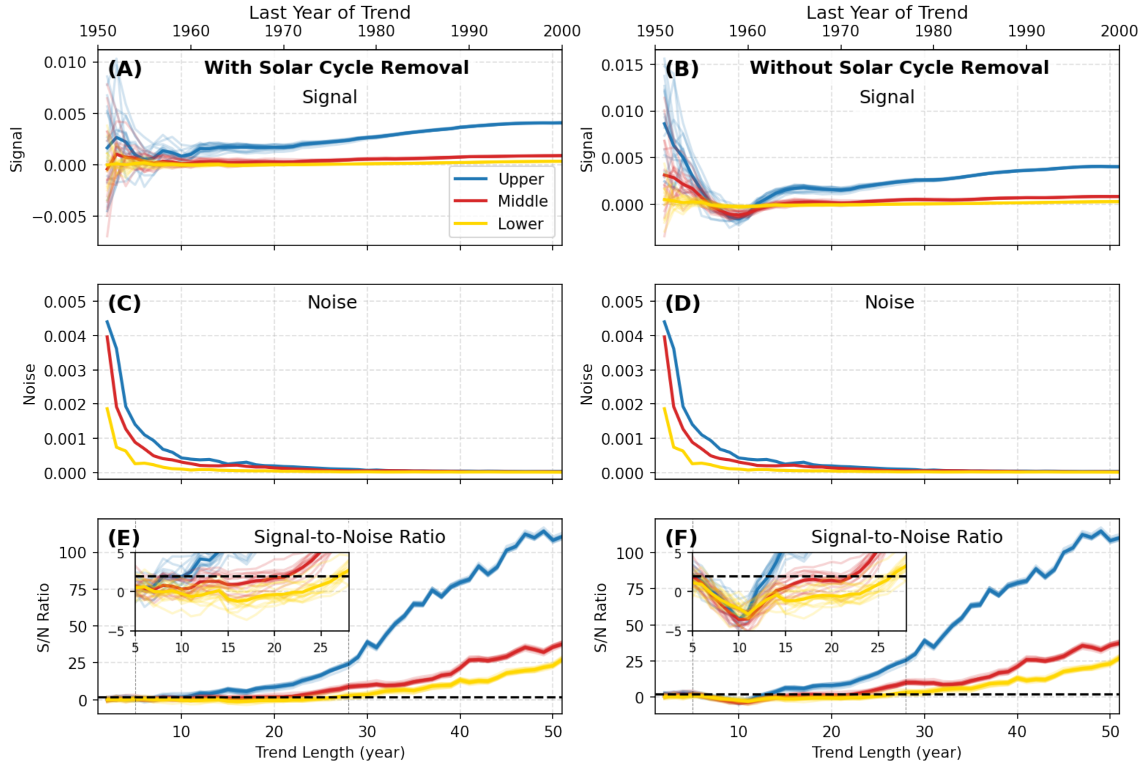

(A) Signal time series, defined as the element-wise uncentered spatial covariance between the fingerprint and the ozone trend pattern in each realization. The trend starts in 1950 and ends incrementally from 1951 to 2000. Lighter lines represent the individual realizations; solid lines denote ensemble means.
(C) Noise time series, defined as the standard deviation of element-wise uncentered spatial covariances between the fingerprint and the residual trend patterns.
(E) Signal-to-noise (S/N) ratio time series. A zoomed-in view is shown for trend lengths of 5 to 28 years.
(B, D, F) Same as panels A, C, and E, respectively, but using ozone data before solar cycle removal.

Figure S6. Emergence times from local signal-to-noise analysis and fingerprinting, and sensitivity to solar cycle removal.

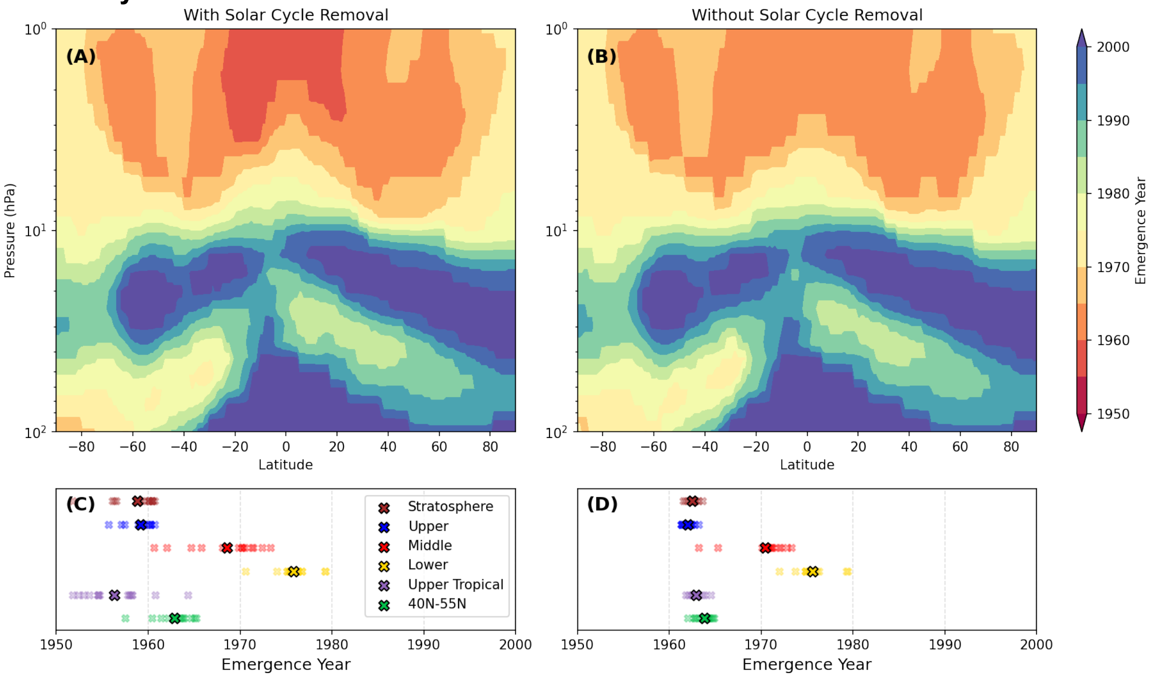

(A) Same as Fig. 5A (local emergence time after solar cycle removal).
(B) Same as panel A, but using WACCM ozone data before solar cycle removal.
(C) Same as Fig. 5B (pattern-based fingerprinting emergence time after solar cycle removal).
(D) Same as panel C, but using ozone data before solar cycle removal.

Figure S7. Emergence times from local signal-to-noise analysis and fingerprinting, and sensitivity to QBO removal.

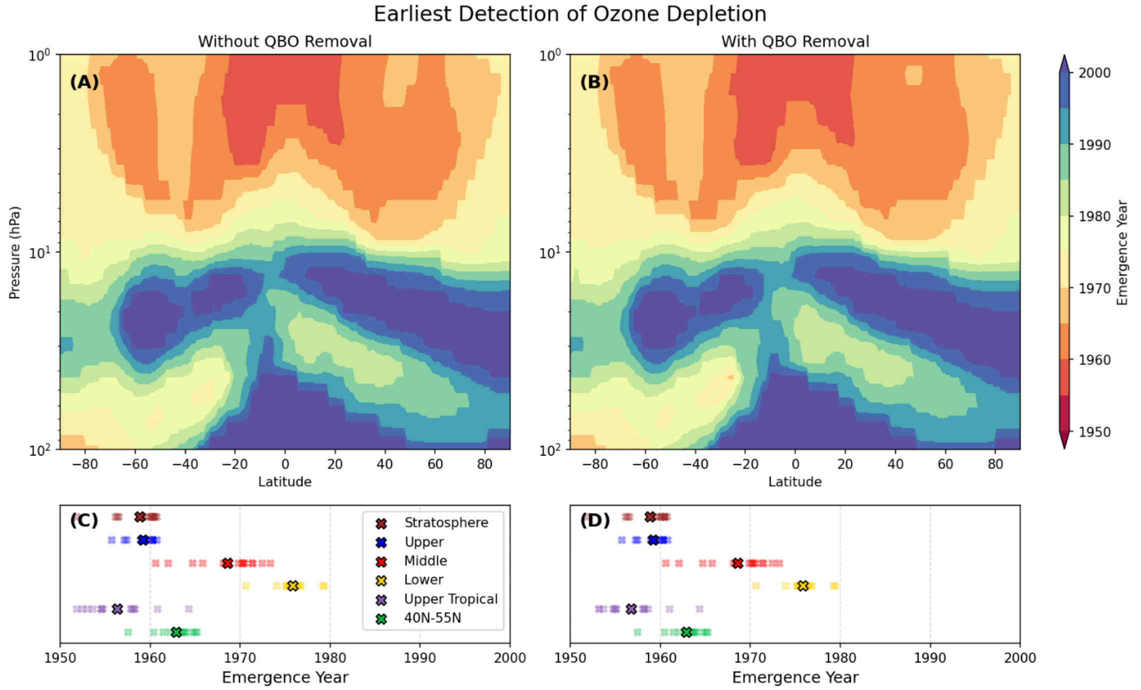

(A) Same as Fig. 5A (local emergence time before QBO removal).
(B) Same as panel A, but using WACCM ozone data after QBO removal.
(C) Same as Fig. 5B (pattern-based fingerprinting emergence time before QBO removal).
(D) Same as panel C, but using ozone data after QBO removal.
